# Observed and expected overall mortality for acute myocardial infarction during the COVID-19 pandemic in Italy: an analysis of nationwide institutional databases

**DOI:** 10.3389/fcvm.2025.1540783

**Published:** 2025-06-06

**Authors:** Leonardo De Luca, Francesco Grippo, Paola D’Errigo, Alessandra Burgio, Stefano Rosato, Barbara Giordani, Giorgia Duranti, Giovanni Baglio

**Affiliations:** ^1^ Department of Cardio-Thoracic and Vascular Medicine and Surgery, S.C. Cardiologia, Fondazione IRCCS Policlinico San Matteo, Pavia, Italy; ^2^ ISTAT—Istituto Nazionale di Statistica, Rome, Italy; ^3^ National Centre for Global Health, Istituto Superiore di Sanità, Rome, Italy; ^4^ Italian National Agency for Regional Healthcare Services, Rome, Italy

**Keywords:** acute myocardial infarction, COVID-19 infection, mortality, administrative database, cohort study

## Abstract

**Aim:**

To carry out a nationwide evaluation of both in- and out-of-hospital mortality for acute myocardial infarction (AMI) during the COVID-19 pandemic period in Italy.

**Methods:**

This was a retrospective cohort study analysing overall mortality for AMI in Italy during the COVID-19 pandemic (March 1st, 2020–December 31st, 2021) and the previous 5 years (January 1st, 2015–February 29th, 2020). To carefully analyze both in- and out-of-hospital mortality for AMI (with or without concomitant COVID-19 infection) we used different institutional administrative sources of national data. Excess mortality related to AMI during the COVID-19 pandemic has been analyzed using the observed/expected ratio (OER).

**Results:**

Over the 5 years pre-pandemic period, 150,299 fatal events related to AMI occurred. During the pandemic, the number of deaths related to AMI was 28,673 in 2020 and declined to 26,688 in 2021. The overall OER was 1.18 [95% confidence intervals (CI): 1.15–1.22] in 2020 and 1.19 (95% CI: 1.15–1.22) while out-of-hospital OER was 1.24 (95% CI: 1.20–1.29) in 2020 and 1.21 (95% CI: 1.16–1.25) during the pandemic. When excluding COVID-19 related deaths, the number of observed in-hospital deaths did not significantly differ from the expected both in 2020 and 2021 while the excess remains unchanged for out-of-hospital mortality.

**Conclusions:**

In this analysis of nationwide institutional administrative databases, we documented an increase in observed mortality compared to the expected during the COVID-19 pandemic in Italy. This mortality increase is mainly attributable to out-of-hospital fatal events and related to concomitant COVID-19 infection for hospitalized AMI patients.

## Introduction

The COVID-19 pandemic had a devastating impact on public health as well as on the organization of the healthcare system (1). Starting from March 2020, Italy was the first country in Europe to be impacted by COVID-19, with over 196,000 deaths and approximately 25 million confirmed cases to date (2, 3). Most documented cases of Covid-19 and linked fatal events occurred in the North of Italy, where the pandemic has primarily afflicted people (2, 3).

The organization of the acute myocardial infarction [AMI] network was negatively affected by COVID-19 pandemic and several studies documented an increase in door-to-balloon time, an increase in mortality with a concomitant decline in hospitalizations during the pandemic (4–10). This reduction in AMI hospitalizations has been attributed to various factors such as lack of access to hospital due to fear of contracting the COVID-19 infection, an increase in out-of-hospital mortality or a general reduction in the incidence of AMI due to environmental factors or lifestyle changes induced by the lockdown period (4–10). At present, a concomitant evaluation of hospitalizations and overall mortality related to AMI during the pandemic period based on national databases is still lacking.

The aim of this analysis, based on nationwide institutional databases, is to describe the observed and expected overall mortality for AMI during the COVID-19 pandemic compared to the pre-pandemic period in Italy. For this purpose, from January 2015 to December 2021, the trend and the changes in hospital admission for AMI, and the overall (in- and out-of-hospital) mortality for AMI (with or without concomitant COVID-19 infection), were analysed and compared.

## Methods

This was a retrospective cohort study that analysed overall mortality for AMI in Italy during the COVID-19 pandemic (March 1st, 2020–December 31st, 2021) and the previous 5 years (January 1st, 2015–February 29th, 2020).

In order to carefully analyze both in- and out-of-hospital mortality for AMI during the observation period we used different institutional administrative sources of national data. Since the registration of information in these databases is mandatory in Italy, the amount of missing data is very residual and there are no missing data in the selected cohort.

The Italian National Registry of Hospital Discharge Records, provided by the Italian Ministry of Health and the National Tax Register, available to the Italian National Program for Outcome Evaluation (PNE-AGENAS) were used as sources of data for the mortality analysis of patients admitted to hospitals for an AMI (11).

We analysed all consecutive patients, resident in Italy, aged 18–100 years, admitted to a public or private Italian hospital from January, 1st 2015 to December, 31st 2021 and reporting a primary diagnosis of AMI [International Classification of Disease, 9th Revision, Clinical Modification (ICD 9 CM) 410] or a secondary diagnosis of AMI with any concomitant AMI complication within the primary diagnosis (ICD-9-CM codes 411, 413, 414, 426, 427, 428, 423.0, 429.5, 429.6, 429.71, 429.79, 429.81, 518.4, 518.81, 780.01, 780.2, 785.51, 799.1, 997.02 and 998.2) [Outcomes evaluation National program (PNE) Ed. 2024; available at https://pne.agenas.it/]. In this cohort, incidence of 1-year all-cause mortality stratified in in-hospital mortality and mortality after discharge have been evaluated. To avoid the inclusion of multiple admissions due to the same event, duplicate records and records concerning transfers of patients to another hospital were excluded. Moreover, following international criteria, multiple admission occurring during 30 days following each index admission were considered as a single AMI episode. Data on patients' risk factors and comorbidities, according to the ICD9-CM codes reported in Supplementary Table S1, were retrieved from either the index admission or the previous 5-year hospitalizations.

Analyses on both in and out-of-hospital deaths were carried out on the Italian National Cause of Death Register (NCoDR) (12), managed by the Italian Institute of Statistics (ISTAT), which collects information on the cause of all deaths occurring in Italy. Causes of death are provided by physicians reporting the sequence of causes directly leading to death and other relevant morbid conditions that may have contributed to death. Out-of-hospital mortality refers to all deaths occurred outside a hospital setting, including pre-hospital and post-discharge events. All conditions reported are classified according to the International Classification of Diseases, 10th Revision (ICD-10) of the World Health Organization (13) with the assistance of the software Iris (https://www.iris-institute.org). The present analyses refer to deaths related to AMI occurred during the study period (from January, 1st 2015 to December, 31st 2021) identified as deaths related to one of the following ICD10 codes: I21.0-I21.9 Acute myocardial infarction, I22.0-I22.9 Subsequent myocardial infarction. The NCoDR also include information on the place of occurrence of death (in or out-of-hospital).

### Statistical analysis

A descriptive analysis of clinical and demographic characteristics of the cohort of hospitalized AMI patients by year of hospitalization has been carried out. Prevalence of risk factors and comorbidities were presented as counts and percentages; age was expressed as the mean ± standard deviation.

Descriptive analysis has also been carried out on the number of deaths due to AMI by place of death (in-hospital vs. out-of-hospital), year of occurrence, geographical area of residence of the deceased (North, Central, South), and age group (≤74 vs. >75 years). In order to assess the geographical area of residence, Italy was divided in three macro-regions: North (Lombardia, Piemonte, Valle d'Aosta, Veneto, Friuli Venezia-Giulia, Trentino Alto-Adige, Liguria and Emilia-Romagna; accounting for a total of 27.4 millions of inhabitants in 2021), Central (Lazio, Toscana, Umbria and Marche; 11.8 millions of inhabitants) and South (Abruzzo, Molise, Puglia, Basilicata, Campania, Calabria, Sicilia and Sardegna; 19.9 millions of inhabitants).

Excess mortality related to AMI during the COVID-19 pandemic has been analyzed using the observed/expected ratio (OER) on the NCoDR. Expected deaths have been estimated with a Poisson model. We chose this model since it is used for count data, such as deaths, which follows a Poisson statistical distribution and it allows to estimate the effect of some explicative variables (sex, age, geographical area, months and year of death in our case) on mortality rates (response variables). This model provides parameters that allow to estimate the expected number of deaths for given values of the explicative variables. In particular, we estimated expected deaths with a 95% confidence interval, using the following explicative variables: sex, age (5-years age groups with the exception of 0 years, 1–4 and 95 and more), area of residence (North, Central, South), year of death, and month of death; the mid-year population was used as offset. We included the month and the year of death in the model since AMI-related mortality shows a seasonal pattern and a declining yearly trend. The model has been fitted on data from January 1st, 2015 to February 29th, 2020 (pre-pandemic period) and the obtained parameters have been used for predicting death for the subsequent pandemic period (March 1st, 2020 to December 31st, 2021). To analyze the different behavior of mortality according to different variables, the model has been stratified by place of death, geographical area, and age group. To take into account the role of COVID-19 in determining excess mortality for AMI, we also estimated expected deaths unrelated to COVID-19 by performing the mentioned Poisson models excluding deaths related to COVID-19, i.e., deaths reporting COVID-19 anywhere on the death certificate (ICD10 codes: U07.1 COVID-19, virus identified; U07.2 COVID-19, virus not identified; U09.9 Post COVID-19 condition, unspecified; U10.9 Multisystem inflammatory syndrome associated with COVID-19, unspecified).

## Results

### Patients hospitalized for AMI

**Figure 1:**
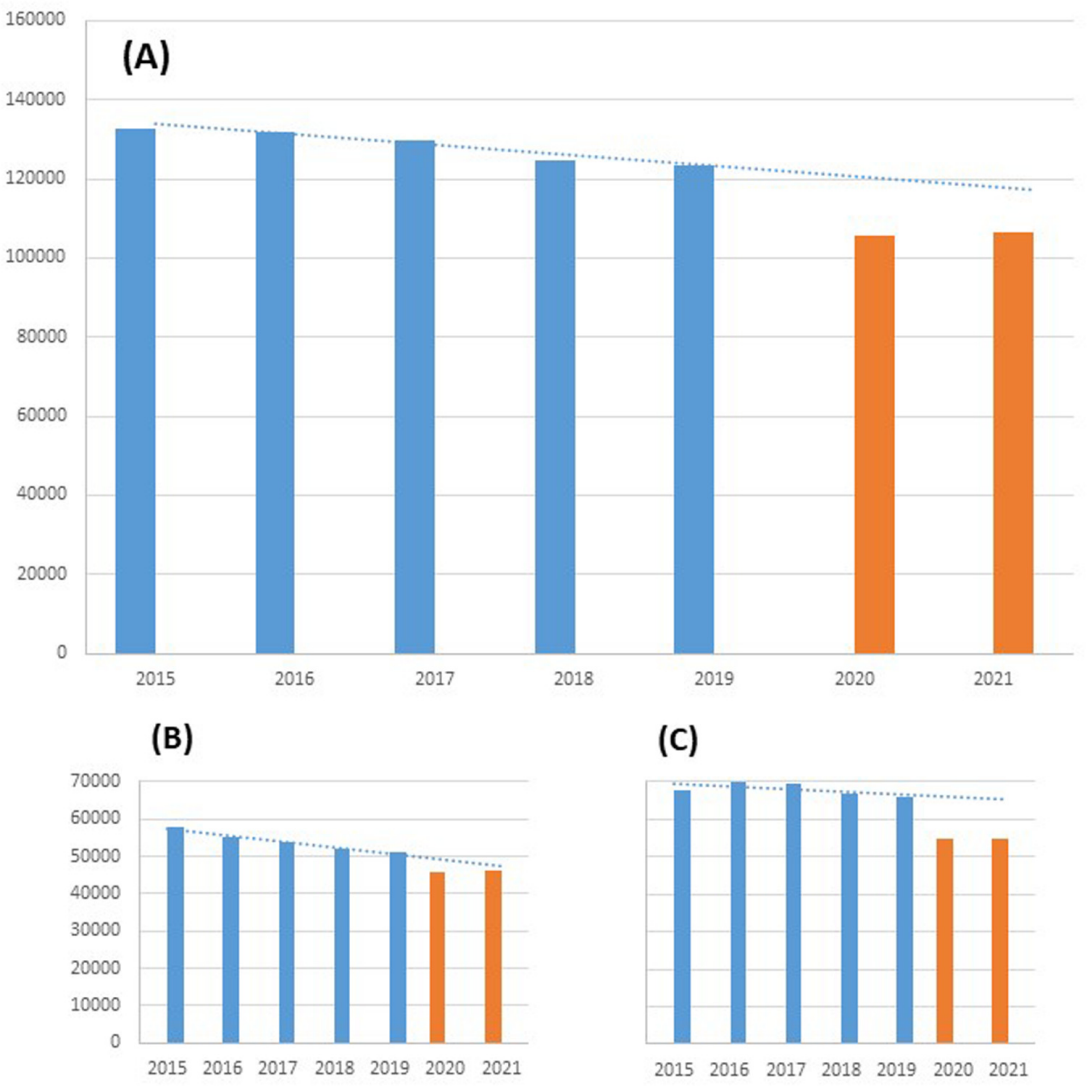
Number of hospitalizations for AMI in Italy before (2015–2019) and during COVID-19 pandemic (2020–2021). **(A)** Overall; **(B)** STEMI; **(C)** NSTEMI.

During the study period, 697,663 AMI patients were admitted to Italian hospitals: 520,439 in the pre-pandemic period (2015–2019) and 177,224 during the COVID-19 pandemic (2020–2021). Trends in the number of hospital admissions for AMI from 2015 to 2021 are shown in Figure 1. In 2020, there was a significant reduction in the number of admissions for AMI, particularly for Non-ST elevation myocardial infarction (NSTEMI). In 2021 a slight increase in hospital admissions for AMI occurred, but not reaching admissions to pre-COVID-19 levels.

Baseline characteristics of patients hospitalized for an AMI event during the study period are shown in Table 1. The mean age of AMI patients admitted to Italian hospitals was 70 years, one third was female, a quarter presented diabetes mellitus and approximately half had a working diagnosis of ST elevation myocardial infarction (STEMI). At 1 year, the rate of mortality for AMI patients who were hospitalized did not substantially changed over time (from 17.1. in the pre-pandemic period to 16.7% in the pandemic period).

**Table 1:** Baseline characteristics of the cohort of patients hospitalized for AMI from 2015 to 2021, by year.

Characteristics and comorbidities	2015 *N* = 106,681	2016 *N* = 106,012	2017 *N* = 105,175	2018 *N* = 101,058	2019 *N* = 101,513	2020 *N* = 87,721	2021 *N* = 89,503
Age, mean ± SD	70.39 ± 13.40	70.3 ± 13.36	70.41 ± 13.24	70.24 ± 13.24	70.18 ± 13.21	69.76 ± 13.01	69.75 ± 13.02
Female	36,640 (34.35)	36,005 (33.96)	35,200 (33.47)	33,657 (33.30)	33,314 (32.81)	27,434 (31.27)	27,614 (30.85)
Diabetes mellitus	26,100 (24.47)	25,966 (24.49)	25,547 (24.29)	24,023 (23.77)	24,022 (23.66)	20,239 (23.07)	20,289 (22.67)
Obesity	4,560 (4.27)	4,611 (4.35)	4,462 (4.24)	4,221 (4.18)	4,277 (4.21)	3,616 (4.12)	4,135 (4.62)
Anemia	8,398 (7.87)	8,509 (8.03)	8,544 (8.12)	8,047 (7.96)	7,982 (7.86)	6,741 (7.68)	6,719 (7.51)
Hypertension	51,107 (47.91)	50,157 (47.31)	49,205 (46.78)	46,425 (45.94)	45,734 (45.04)	39,151 (44.63)	38,725 (43.27)
Prior AMI	17,982 (16.86)	17,433 (16.44)	16,770 (15.94)	15,715 (15.55)	15,411 (15.18)	12,486 (14.23)	11,984 (13.39)
History of HF	26,513 (24.85)	25,556 (24.11)	25,302 (24.06)	23,964 (23.71)	23,965 (23.60)	19,621 (22.37)	20,392 (22.78)
Cerebrovascular diseases	12,850 (12.05)	12,403 (11.7)	11,853 (11.27)	10,753 (10.64)	10,303 (10.15)	8,121 (9.26)	7,649 (8.55)
Vascular diseases	9,075 (8.51)	8,911 (8.41)	8,438 (8.02)	7,849 (7.77)	7,578 (7.46)	6,215 (7.08)	5,962 (6.66)
COPD	11,282 (10.58)	10,308 (9.72)	9,959 (9.47)	8,775 (8.68)	8,576 (8.45)	6,386 (7.28)	5,885 (6.58)
CKD	14,530 (13.62)	14,572 (13.75)	14,192 (13.49)	13,144 (13.01)	12,985 (12.79)	10,641 (12.13)	10,316 (11.53)
Malignant tumor	9,097 (8.53)	8,554 (8.07)	8,827 (8.39)	8,358 (8.27)	8,166 (8.04)	6,975 (7.95)	7,024 (7.85)
Prior CABG	6,652 (6.24)	6,598 (6.22)	6,322 (6.01)	5,787 (5.73)	5,640 (5.55)	4,476 (5.10)	4,178 (4.67)
Prior PCI	18,637 (17.47)	18,734 (17.67)	18,792 (17.87)	18,598 (18.40)	18,639 (18.36)	15,555 (17.73)	15,141 (16.92)
Geographic area							
North	48,866 (45.81)	48,784 (46.02)	47,970 (45.61)	46,169 (45.69)	46,730 (46.03)	40,444 (46.11)	41,909 (46.82)
Central	21,807 (20.44)	21,485 (20.27)	20,946 (19.92)	20,160 (19.95)	19,923 (19.62)	17,053 (19.44)	16,939 (18.93)
South	36,005 (33.75)	35,740 (33.71)	36,259 (34.47)	34,729 (34.37)	34,878 (34.35)	30,224 (34.45)	30,655 (34.25)
Type of AMI							
STEMI	45,356 (42.52)	43,578 (41.11)	43,076 (40.96)	41,150 (40.72)	41,521 (40.90)	37,132 (42.33)	38,042 (42.50)
NSTEMI	55,750 (52.26)	57,146 (53.91)	57,300 (54.48)	55,097 (54.53)	55,050 (54.22)	46,103 (52.56)	46,757 (52.24)
Unknown	5,574 (5.22)	5,285 (4.99)	4,798 (4.56)	4,799 (4.75)	4,959 (4.88)	4,486 (5.11)	4,703 (5.25)
COVID-19 infection	–	–	–	–	–	2,174 (2.19)	2,503 (2.50)
1-year mortality	19,198 (18.00)	18,819 (17.75)	17,979 (17.07)	16,698 (16.52)	16,541 (16.29)	14,997 (17.10)	14,593 (16.23)

AMI, acute myocardial infarction; CABG, coronary artery bypass grafting; COPD, chronic obstructive pulmonary diseases; CKD, chronic kidney disease; HF, heart failure; NSTEMI, non ST-elevation myocardial infarction; PCI, percutaneous coronary intervention; STEMI, ST-elevation myocardial infarction.

### Analysis of mortality for AMI

Considering all deaths related to AMI over the pre-pandemic period (NCoDR), 150,299 fatal events occurred (4.7% of total deaths), corresponding to an annual average of 30,060 deaths (Table 2). During the pandemic, the number of deaths related to AMI was 28,673 in 2020 and declined to 26,688 in 2021 (3.8% of total deaths in both 2020 and 2021). On average, in 2015–2019, 39.4% of AMI deaths occurred in hospitals, but this percentage dropped to 36.1% in 2020 with a slight increase in 2021 (37.9%).

**Table 2:** Number of deaths due to AMI in the Italian population and results of the Poisson models (overall and stratified).

	Year	Overall deaths			In-hospital deaths			Out-of-hospital deaths			In-hospital deaths without COVID-19			Out-of-hospital deaths without COVID-19		
		Observed	Observed/ expected	CI 95%	Observed	Observed/ expected	CI 95%	Observed	Observed/ expected	CI 95%	Observed	Observed/ expected	CI 95%	Observed	Observed/ expected	CI 95%
Overall																
	2015–2019	150.299	1.00	0.97-1.03	59.242	1.00	0.96-1.04	91.057	1.00	0.97-1.03	59.242	1.00	0.96–1.04	91.057	1.00	0.97–1.03
	2020	28.673	1.18	1.15–1.22	10.349	1.08	1.04–1.13	18.324	1.24	1.20–1.29	9.252	0.97	0.93–1.01	18.022	1.22	1.18–1.27
	2021	26.688	1.19	1.15–1.22	10.127	1.14	1.09–1.20	16.561	1.21	1.16–1.25	9.083	1.03	0.98–1.08	16.265	1.19	1.14–1.23
Stratified by geographical area																
North	2015–2019	73.008	1.00	0.96–1.04	31.660	1.00	0.95–1.06	41.348	1.00	0.95–1.05	31.660	1.00	0.95–1.06	41.348	1.00	0.95–1.05
	2020	14.460	1.24	1.19–1.29	5.770	1.15	1.09–1.22	8.690	1.30	1.24–1.37	5.006	1.00	0.94–1.06	8.477	1.27	1.21–1.34
	2021	13.132	1.23	1.18–1.27	5.482	1.19	1.12–1.27	7.650	1.24	1.18–1.31	4.878	1.06	1.00–1.13	7.479	1.22	1.15–1.28
Center	2015–2019	28.927	1.00	0.94–1.06	12.594	1.00	0.92–1.09	16.333	1.00	0.93–1.08	12.594	1.00	0.92–1.09	16.333	1.00	0.93–1.08
	2020	5.288	1.12	1.05–1.18	1.975	0.98	0.90–1.08	3.313	1.21	1.12–1.31	1.811	0.91	0.83–0.99	3.276	1.20	1.10–1.29
	2021	4.943	1.11	1.04–1.19	1.972	1.06	0.96–1.16	2.971	1.15	1.06–1.25	1.809	0.97	0.88–1.07	2.920	1.13	1.04–1.23
South	2015–2019	48.364	1.00	0.96–1.05	14.988	1.00	0.92–1.08	33.376	1.00	0.95–1.06	14.988	1.00	0.92–1.08	33.376	1.00	0.95–1.06
	2020	8.925	1.14	1.09–1.19	2.604	1.03	0.95–1.12	6.321	1.18	1.12–1.25	2.435	0.97	0.89–1.05	6.269	1.17	1.11–1.24
	2021	8.613	1.17	1.12–1.23	2.673	1.11	1.02–1.22	5.940	1.19	1.12–1.27	2.396	1.00	0.92–1.09	5.866	1.18	1.11–1.25
Stratified by age group																
≤74 years	2015–2019	46.327	1.00	0.96–1.04	15.582	1.00	0.93–1.08	30.745	1.00	0.95–1.06	15.582	1.00	0.93–1.08	30.745	1.00	0.95–1.06
	2020	9.219	1.20	1.14–1.25	2.970	1.12	1.04–1.21	6.249	1.23	1.16–1.30	2.659	1.01	0.93–1.09	6.183	1.21	1.15–1.28
	2021	8.786	1.21	1.15–1.27	3.030	1.20	1.11–1.31	5.756	1.20	1.13–1.28	2.726	1.09	1.00–1.18	5.653	1.18	1.11–1.25
>75 years	2015–2019	103.972	1.00	0.97–1.03	43.660	1.00	0.96–1.04	60.312	1.00	0.97–1.03	43.660	1.00	0.96–1.04	60.312	1.00	0.97–1.04
	2020	19.454	1.18	1.14–1.21	7.379	1.07	1.02–1.12	12.075	1.25	1.21–1.30	6.593	0.96	0.91–1.00	11.839	1.23	1.18–1.28
	2021	17.902	1.17	1.14–1.21	7.097	1.12	1.07–1.17	10.805	1.21	1.16–1.26	6.357	1.00	0.96–1.05	10.612	1.19	1.14–1.24

**Figure 2:**
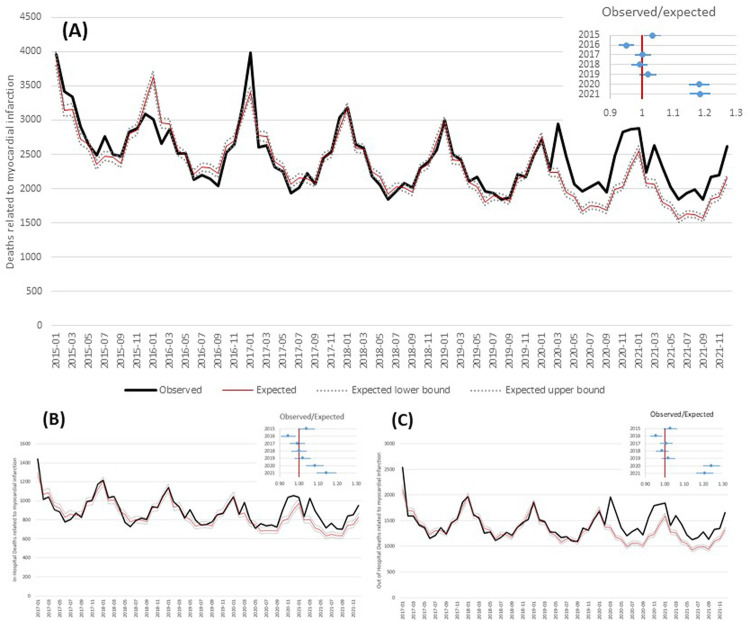
Observed and expected (based on Poisson model) deaths related to acute myocardial infarction by month and observed/expected ratio by year. **(A)** Overall; **(B)** in-hospital deaths; **(C)** out-of-hospital deaths.

The comparison between observed and expected overall deaths (Figure 2) shows an excess of AMI deaths from March 2020 through December 2021, with peaks in March, April, and November 2020 and smaller peaks in March, April, and December 2021. In general, the OER was 1.18 [95% confidence intervals (CI): 1.15–1.22] in 2020 and 1.19 (95% CI: 1.15–1.22) in 2021 (Figure 2A). In-hospital deaths showed a lower OER, although significantly higher than 1: the value in 2020 was 1.08 (95% CI: 1.04–1.13) in 2020 and 1.14 (95% CI: 1.09–1.20) in 2021 (Figure 2B). Out-of-hospital OER was 1.24 (95% CI: 1.20–1.29) in 2020 and 1.21 (95% CI: 1.16–1.25) in 2021 (Figure 2C). This increase in out-of-hospital mortality was evident in all geographical areas and in both age groups (Table 2).

**Figure 3:**
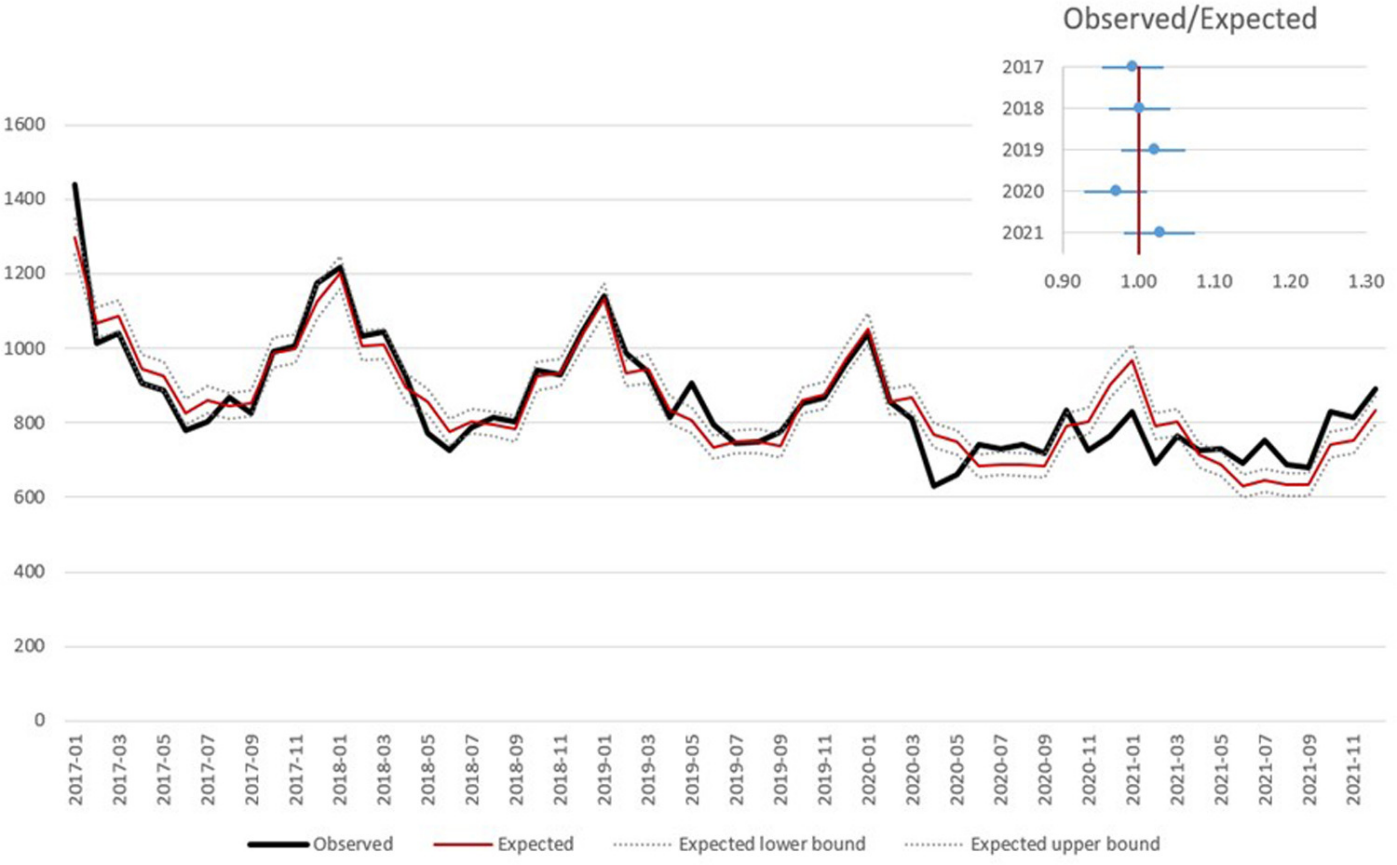
Observed and expected (based on Poisson model) in hospital deaths related to AMI by month and observed/expected ratio by year, excluding deaths with concomitant COVID-19.

By excluding COVID-19 related deaths from the analysis of the in-hospital mortality, the number of observed deaths did not significantly differ from the expected both in 2020 and 2021, in all geographical areas and in both age groups; conversely, the number of observed deaths is confirmed significantly higher than expected when considering out-of-hospital mortality (North: OER-2020 = 1.27, OER-2021 = 1.22; Center: OER-2020 = 1.20, OER 2021 = 1.13; South: OER-2020 = 1.17, OER-2021 = 1.18; ≤74 years: OER-2020 = 1.21, OER-2021 = 1.18; >75 years: OER-2020 = 1.23, OER-2021 = 1.19) (Table 2; Figure 3).

## Discussion

The key insights of our analysis are the following: (1) During the COVID-19 pandemic, we observed a reduction in the number of patients hospitalized for AMI who presented a similar 1-year mortality compared to the pre-pandemic period; (2) Compared to the expected rates calculated on trends over the previous 5 years, a marked increase in out-of-hospital mortality was observed during pandemic in Italy; (3) Excluding deaths related to COVID-19 infection, the observed and expected in-hospital mortality rates overlap, regardless of the geographical area and age group.

As already documented in several retrospective studies (6–10, 14–18), in this analysis based on nationwide institutional databases with whole population coverage, we have confirmed the reduction in hospitalizations for AMI during the pandemic period. However, the clinical characteristics and mortality at 1 year of follow up of patients hospitalized for AMI remained relatively unchanged during the observation period of our analysis, suggesting that the increase in overall mortality observed during the pandemic cannot be directly correlated to poorer acute care for AMI nor to a lower adoption of the secondary prevention strategies adopted in AMI patients hospitalized during the pandemic compared to the previous period.

There are two basic theories that help explain the decline in AMI hospital admissions during pandemic. The first is that fewer AMI were induced by the lifestyle adjustments (e.g., reducing stress, reducing air pollution) brought about by the epidemic and lockdown. The second is that people's decision to visit a hospital while experiencing AMI symptoms may have been hampered by their concern of spreading the illness and their misreading of the government's “stay-at-home” campaign. In this regard, analyzing the observed and expected incidence of mortality in the pandemic period, an increase in fatal events compared to expected was observed, especially for out-of-hospital mortality, while in-hospital deaths remained more similar to the expected in all geographical areas and age groups. This confirms that the total increase in AMI mortality observed during the pandemic is mainly attributable to out-of-hospital mortality (probably due to the lack of access to hospital, especially of patients with NSTEMI for fear of contracting the COVID-19 infection), while the in-hospital management of AMI has remained almost unchanged over time with short-term and long-term outcomes comparable to the pre-pandemic period.

Previous research regarding the outcomes of AMI patients hospitalized during the pandemic revealed inconsistent findings (4–6, 19–22). An increase in hospital mortality was shown by a large retrospective registry of STEMI patients receiving coronary revascularization across European centers (4–6). This result was not supported by other research (19–22), where the early death of AMI patients hospitalized during the pandemic was consistent with prior years' rates following correction for possible confounding variables. Even our nationwide analysis of national databases supported this conclusion. Although potential confounding factors, such as healthcare system strain, changes in AMI treatment protocols, and delays in seeking medical care cannot be excluded, it appears that during the pandemic, a well-established and structured AMI network was able to perform well without causing appreciable changes in mortality (7, 14). In this regard, according to Eurostat database (https://ec.europa.eu/eurostat/en/), in 2021 Italy had a standardized mortality rate for AMI of 24.4 deaths per 100,000 individuals, lower than the EU27 average (37.4 deaths per 100,000 individuals). Italian value was among the lowest within Europe, smaller rates were observed in France (17.6) and Denmark (19.7). Such situation was similar also in the prepandemic period: in 2019 the Italian rate was 26.2 compared to 37.5 of EU27.

Unfortunately, the same consideration cannot be applied to AMI patients with confirmed COVID-19 infection. Indeed, even in other series, patients with AMI and concomitant COVID-19 infection presented a higher in-hospital mortality (22–24). Accordingly, in our analysis the presence of a COVID-19 infection appears to be the major determinant of mortality, especially for hospitalized AMI patients, regardless of the geographical area and age. In fact, by eliminating the deceased patients with a confirmed diagnosis of COVID-19, even the observed in-hospital mortality for AMI returns to expected levels.

The current investigation has the following major strengths: (i) data were extracted from institutional databases with total population coverage including consecutive AMI patients admitted to every Italian hospital, both public and private, and assessed every death related to AMI that occurred during the study period in Italy; (ii) the analysis was not limited to pandemic period, but the historical series starting from January 2015 was reported; (iii) data on in- and out-of-hospital mortality due to AMI were available (considering both those with and without concomitant COVID-19 infection); (iv) data of hospitalized AMI patients were enriched with 1-year follow-up mortality to depict the whole spectrum of the consequences of the COVID-19 pandemic on early AMI management.

### Limitations

There are several limitations of using an administrative health claims database. One is the lack of specific clinical information may have affected the accuracy of the diagnosis, severity and risk stratification of AMI. Indeed, for hospital discharge records, some prognostic data, such as vital signs, instrumental parameters, time to reperfusion and procedural detail were not available. We also considered multiple hospital admissions occurring within 30 days as a single AMI event, generating a potential bias. In addition, it was not possible to control for confounders such as the presence of cardiovascular risk factors, when estimating in-hospital and out-of-hospital mortality, due to the lack of information about the morbidity status of the patients in the NCoDR. Another limitation of the mentioned database is the deficiencies in the ICD-9 CM code descriptions to provide comprehensive data on in-hospital complications and cause of death. In this regard, it was not feasible to completely rule out conditions associated with COVID-19 infection that may have produced an uncontrolled bias due to their association with mortality. Finally, we cannot determine the extent to which misclassification and coding errors may be present.

## Conclusions

In the present analysis of nationwide institutional administrative databases, we documented an increase in observed mortality compared to the expected rate during the COVID-19 pandemic in Italy. This increase in mortality is mainly attributable to out-of-hospital fatal events and related to concomitant COVID-19 infection for hospitalized AMI patients.

## Data Availability

The datasets presented in this article are not readily available as they are not publicly available. Requests for access to the datasets should be directed to the ISTAT Contact Centre (https://contact.istat.it/s/?language=it) for the cause-of-death dataset and to AGENAS (baglio@agenas.it) for the Hospital Discharge Records dataset.
