# Supplementary material for: Observed and expected overall mortality for acute myocardial infarction during the COVID-19 pandemic in Italy: an analysis of nationwide institutional databases
Source: Front Cardiovasc Med. 2025 Jun 6;12:1540783. doi: 10.3389/fcvm.2025.1540783 (PMC12179071; doi:10.3389/fcvm.2025.1540783)
Supplement: Supplementary file 1 [file Table1.pdf]

SUPPLEMENTARY TABLE 1. ICD9-CM codes used to retrieve information on risk factors and comorbidities

	ICD9 Code	
	**Index admission**	**Previous admission**
Hypertension		401-405
Diabetes mellitus		250
Prior AMI	412	410, 412
Cerebrovascular diseases	433, 437, 438	430-434, 436-438
Vascular diseases	440-448(except 441.1, 441.3, 441.5, 441.6, 444), 557.1	440-448, 557
History of HF		428
CKD		426, 427
Anemia	280-284, 285 (except 285.1)	280-284, 285 (except 285.1)
Obesity	278.0	278.0
COPD		491, 492, 494, 496
Malignant tumor	V10	140.0–208.9, V10
Previous CABG	V45.81	36.1, V45.81
Previous PCI	V45.82	00.66, 36.0, V45.82
Type of AMI		
STEMI	410.0-410.6	
NSTEMI	410.7	
Unknown	410.8, 410.9	
COVID-19 infection	043, 078.89, 079.82, 480.3, 480.4, 484.8, 518.9, 519.7, V01.79, V01.82, V01.85, V07.0, V12.04, V71.83, V71.84	

Abbreviations: AMI: acute myocardial infarction; CABG: coronary artery bypass grafting; COPD: chronic obstructive pulmonary diseases; CKD: chronic kidney disease; HF: heart failure; NSTEMI: Non ST-elevation myocardial infarction; PCI: percutaneous coronary intervention; STEMI: ST-elevation myocardial infarction
